# Correction to “Quadruple‐editing of the MAPK and PI3K pathways effectively blocks the progression of KRAS‐mutated colorectal cancer cells”

**DOI:** 10.1111/cas.70145

**Published:** 2025-07-14

Wang Z, Kang B, Gao Q, et al. Quadruple‐editing of the MAPK and PI3K pathways effectively blocks the progression of KRAS‐mutated colorectal cancer cells. *Cancer Sci*. 2021;112:3895–3910. https://doi.org/10.1111/cas.15049

In the above article, Figure 6(F) is incorrect. The correct image is shown below:

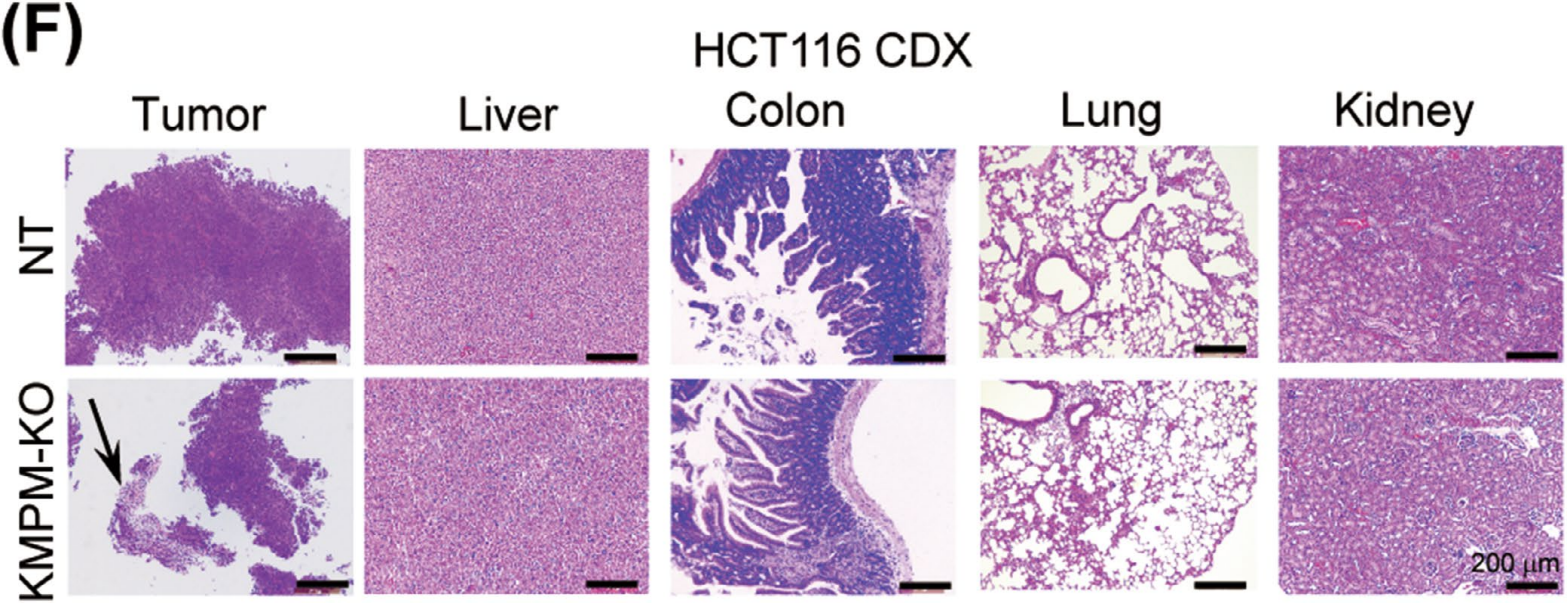

We apologize for this error.

